# Spatio-Temporal Brain Dynamic Differences in Fluid Intelligence

**DOI:** 10.3389/fnhum.2022.820780

**Published:** 2022-03-03

**Authors:** Nadja Tschentscher, Paul Sauseng

**Affiliations:** ^1^Research Unit Biological Psychology, Department of Psychology, Ludwig Maximilian University Munich, Munich, Germany; ^2^Research Unit Clinical Psychology, Department of Psychiatry and Psychotherapy, Ludwig Maximilian University Munich, Munich, Germany

**Keywords:** executive functions (EFs), fluid intelligence, EEG, fronto parietal network, goal directed behaviour

## Abstract

Human fluid intelligence is closely linked to the sequential solving of complex problems. It has been associated with a distributed cognitive control or multiple-demand (MD) network, comprising regions of lateral frontal, insular, dorsomedial frontal, and parietal cortex. Previous neuroimaging research suggests that the MD network may orchestrate the allocation of attentional resources to individual parts of a complex task: in a complex target detection task with multiple independent rules, applied one at a time, reduced response to rule-critical events across the MD network in lower fluid intelligence was observed. This was in particular the case with increasing task complexity (i.e., larger sets of rules), and was accompanied by impairment in performance. Here, we examined the early spatiotemporal neural dynamics of this process in electroencephalography (EEG) source analyses using a similar task paradigm. Levels of fluid intelligence specifically predicted early neural responses in a left inferiorparietal MD region around 200–300 ms post stimulus onset. Evoked source amplitudes in left parietal cortex within this early time window also correlated with behavioural performance measures. Like in previous research, we observed impaired performance in lower fluid intelligence with increasing number of task rules. This links fluid intelligence to a process of attentional focus on those parts of a task that are most critical for the current behaviour. Within the MD system, our time re-resolved measures suggest that the left parietal cortex specifically impacts on early processes of attentional focus on task critical features. This is novel evidence on the neurocognitive correlates of fluid intelligence suggesting that individual differences are critically linked to an early process of attentional focus on task-relevant information, which is supported by left parietal MD regions.

## Introduction

In a complex environment, human behaviour depends to a large extent on the ability to think logically and to solve problems in the absence of task-specific knowledge (Duncan, 2013). Those skills are operationalised by fluid intelligence, a core measure of psychometric assessment (Carpenter et al., 1990). Fluid intelligence can be measured with complex, multistep tasks involving novel rules (Raven et al., 1988). With lower fluid intelligence, errors increase with the number of task rules, even if the rules are known and can be remembered correctly (Duncan et al., 2008; Bhandari and Duncan, 2014), suggesting a broad inability to foreground the correct part of a complex rule set. In a complex target detection task with a varying number of independent rules, applied one at a time in successive task epochs, it has been shown that although only one rule was applied at a time, increasing task complexity (i.e., either 2 or 4 rules) impaired performance in participants of lower fluid intelligence (Tschentscher et al., 2017). This suggests that lower fluid intelligence is reflected in the inability to focus on the correct part of a complex rule set. Conversely, achieving focus on the correct cognitive operation of each task stage is a feature of high fluid intelligence (Duncan, 2013).

Lesion and functional magnetic resonance imaging (fMRI) research links fluid intelligence to a specific set of frontal and parietal regions, here called the multiple-demand (MD) network, comprising regions of lateral frontal, insular, dorsomedial frontal, and parietal cortex (Prabhakaran et al., 1997; Duncan et al., 2000; Gray et al., 2003; Woolgar et al., 2010; Cole et al., 2015). Using fMRI, a novel-rule paradigm previously specified this link, showing that lower fluid intelligence is not only associated with higher error rates when more rules had to be configured for behaviour, but also reduced responses in MD regions (Tschentscher et al., 2017).

While similar fMRI response patterns have been observed across MD regions by some researchers (Duncan and Owen, 2000; Duncan, 2010), others have found highly specific responses of particular MD regions associated with different levels of fluid intelligence: during a complex reasoning task, a stronger upregulation of fMRI responses in posterior regions was observed for highly intelligent in comparison to average intelligent adolescents in correlation with task performance (Lee et al., 2006). In an fMRI based meta-analysis, the dorsal attention network, which overlaps with the parietal MD network, represented the most consistent correlate of fluid intelligence in abstract reasoning tasks (Santarnecchi et al., 2017a). It has been also claimed that the MD system is divided into components for introspective processing, related to the default mode network, and parts for the regulation of attention, related to the dorsal attention network (Dixon et al., 2018). Since the allocation of attentional resources to relevant task features is a key component of high fluid intelligence (Duncan, 2013), individual differences might be particularly driven by parietal regions of attentional regulation.

Critically, there is yet little evidence on the spatiotemporal brain dynamics underlying fluid intelligence across MD regions. Time-resolved information is needed to tell whether frontal and parietal parts operate sequentially or in parallel across time. In combined electroencephalography (EEG) and magnetoencephalography (MEG) source measurements, differential activation dynamics have been observed across MD regions during early stages (200–300 ms) of complex cognition (Tschentscher and Hauk, 2015, 2016), and evidence from EEG evoked potentials suggests that N2 and P3 components are specifically predictive for individual differences in fluid intelligence (Amin et al., 2015; Luo and Zhou, 2020; Rico-Picó et al., 2021). More specifically, the P3b component, a positive wave that peaks around 250–300 ms at parietal cortical regions, has been associated with individual differences in fluid intelligence (Amin et al., 2015; Teixeira-Santos et al., 2020). The P3b has been linked to the allocation of attentional resources to relevant task features, while the P3a component, that peaks around 250–280 ms at frontal sources, has been linked to stimulus-driven involuntary attention mechanisms and processing of novelty (Polich, 2007).

Here, we examined the spatiotemporal neural dynamics of fluid intelligence within MD regions in EEG source models. A similar task paradigm was used as in previous fMRI research (Tschentscher et al., 2017). Multiple independent rules had to be applied one at a time in successive task epochs. Participants either had to memorize 2 or 4 novel rules in different experimental runs. Thus, while the complexity of each task epoch was held constant across runs, the demands to configure individual rules in a mental task model varied. Configuring multiple independent rules for behaviour at the same time is a key component of fluid intelligence, and is measured in standardised tests of fluid intelligence (Cattell and Cattell, 1960; Raven et al., 1988).

We predicted that participants with average fluid intelligence, in contrast to highly intelligent individuals, show weaker MD activity for the rule in operation, especially in the more complex 4-rule context. Furthermore, we hypothesised that individual differences in fluid intelligence first occur in dorsal MD regions involved in processes of attentional focus on task-critical information, as related to the P3b component.

## Materials and Methods

### Participants

EEG was recorded from 43 volunteers after giving written informed consent. Three participants were excluded from analyses due to too many blinks, horizontal eye movements, or incomplete data sets. A power analysis based on effect sizes observed in a recent fMRI study on individual differences in fluid intelligence within the MD system (Tschentscher et al., 2017) suggested that a minimum of 20 subjects per IQ-group was required (*f* = 0.20; alpha = 0.05, power = 90%). Participants were divided into groups of lower and higher fluid intelligence based on a median-split analysis on the scores of the cultural-fair intelligence test (Cattell and Cattell, 1960). The median at IQ-score 109 (raw-score of 35) divided the sample into a group of participants with lower IQ scores, here called the “average-IQ group,” (*n*_average–IQ_ = 20, mean = 97, SD = 7), and a group with higher IQ-scores, the “high-IQ group” (*n*_high–IQ_ = 20, mean = 125, SD = 11). The mean age of the average-IQ participants was 30 years (SD = 7), the mean age of the high-IQ participants was 30 years as well (SD = 8). Five out of 20 average-IQ participants were male and 15 female, and seven out of 20 high-IQ participants were male and 13 female.

All participants had normal or corrected to normal vision and were not affected by neurological or psychiatric disorders. Right-handedness of all participants was confirmed by the Edinburgh-Handedness-Scale (Oldfield, 1971) (mean average-IQ participants = 84, SD = 15; mean high-IQ participants = 88, SD = 16). The level of education was assessed by using seven categories: 0 = no school degree, 1 = basic school degree (after 10 years of schooling), 2 = apprenticeship, 3 = A-levels, 4 = bachelor’s degree, 5 = master’s degree, 6 = doctoral degree, and 7 = professorship. The level of education did not differ significantly between the IQ groups (Mann-Whitney-*U*-Test: *p* = 0.107); median level of education average-IQ = 3, median level of education high-IQ = 3. Participants were financially compensated for participation. The study was approved by the University of Munich Ethics Committee.

### Experimental Paradigm

**FIGURE 1:**
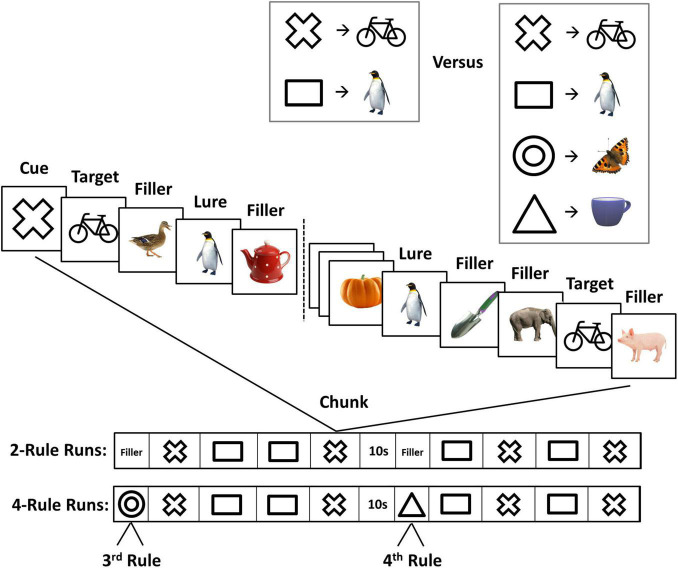
The experimental paradigm. *Top*, before each experimental run, participants learned two or four novel cue-target associations (rules). *Middle*, in each task chunk (29 s), a cue preceded a sequence of animate and inanimate objects. The cue indicated the target in the current chunk. Other images were fillers and lures (images associated with a different cue). *Bottom*, sequence of task chunks in 2- and 4-rule runs.

The design of the task was very similar to the one used in a previous fMRI study (Tschentscher et al., 2017; Figure 1). For each 4.5 min run of the experiment, participants were asked to memorize two or four novel associations between geometric figures (cues) and animate or inanimate objects (targets) (Figure 1, upper right). The run was then divided into a series of chunks, each beginning with the appearance of a single cue from the memorised set, followed by a series of pictures presented at 1/s (0.7 s on, 0.3 s interstimulus interval). The task was to press a key with the right hand whenever the specified target appeared. Within each chunk, the cued target appeared twice, as did the image associated with the other cue (lure, to be ignored). Remaining pictures were fillers, which were never used as targets, with the number of fillers between each critical event (cue, target, or lure) jittered between two and eight. Each run was divided into two halves by a 10 s pause in the middle. Each half-run contained four 29 s chunks, two for each rule (A and B) in ABAB, BABA, ABBA, or BAAB order (Figure 1).

There was a brief period (15 s) of filler pictures before the first chunk of each 2-rule half-run, again presented at 1/s, which the participant simply watched while awaiting the first cue (for example, a sequence of a run was “15s-A-B-B-A-15s-B-B-A-A”). For 4-rule runs, two extra rules were used only once each, one for the 15 s period at the start of the first half-run, the other for a similar period at the start of the second half-run. In 4-rule runs, these 15 s periods consisted of the cue, 12 fillers, and, randomly placed within these, a single target and a single lure. To ensure the comparability of 2- and 4-rule data, analysis focused just on the eight main chunks of each run, discarding the initial period of each half-run (fillers for 2-rule runs; the two extra rules for 4-rule runs). Thus, analyses of 2- and 4-rule runs focussed only on the task *execution* of either two rules. However, 2- and 4-rule runs differed in the number of potentially relevant rules that people had to *consider* for behaviour.

Across the whole experiment there were 12 runs: six 2-rule runs and six 4-rule runs. Different cues and targets were used for each run. Within each run, half of the targets were animate and the others were inanimate. Half of the filler items on each run were animate, the others were inanimate. Before the beginning of each run, two slides were presented. The first slide indicated the cue–target associations, and stated “please memorize the following associations.” The second slide asked participants to recall the images associated with the previously learned cues. The order of cues presented for the memory check was randomised and did not match the order of presentation on the initial instruction slide. Participants stated their responses during the memory check verbally and were allowed to see the initial instruction slide again in case they were not confident. At the end of each run, another memory check slide was presented, asking participants to recall the images associated with the randomly presented cues.

### Stimuli and Visual Display

Stimuli were presented using MATLAB Psychtoolbox-3^1^, which was synchronised with the BrainVision Recorder 2.0.4 (BrainProducts^®^), on a 22-inch Samsung S22C450 monitor with a resolution of 1280 × 1024 and a 75 Hz refresh rate. The monitor was placed centrally at 80 cm distance from the observer. Stimuli were colour images of animals and objects drawn from multiple open-source visual image databases with a visual angle ranging from 3° to 4.7°. The visual display included a white background and a black fixation cross that was presented in the 0.3 s inter-stimulus interval.

### Electroencephalography Data Acquisitions

EEG was recorded while participants performed the task. EEG was registered from 60 scalp locations with Ag–AgCl electrodes arranged in an electrode cap (Easycap) according to the extended 10-10-system, using a BrainAmp DC amplifier (BrainProducts). The EOG was recorded through electrodes placed above the left eye (vertical EOG) and at the outer canthi (horizontal EOG). As a recording reference, a ring-electrode was placed on the tip of the nose. The ground electrode was placed at position FPz. Electrode impedances were kept below 15 kΩ. EEG data were digitised at 1,000 Hz in a frequency range between 0.016 and 250 Hz. A notch filter was applied at 50 Hz. EEG data were inspected visually, and interpolation of EEG channels was applied. Eye movement artifacts in EEG data were removed by an independent component analysis, run in MNE Python software v0.21.0 (Gramfort et al., 2013). This was applied to continuous data to identify and subsequently remove components with a time course correlating with eye movement artefacts. Data were offline band-pass filtered between 0.1 and 40 Hz before averaging. For each condition (targets, lures, and fillers of 2- and 4-rule runs, respectively) epochs from −300 to 800 ms after onset of the stimulus were averaged by using the MNE Python software v0.21.0. Trials were rejected during averaging using the “autoreject” algorithm implemented in MNE Python (Jas et al., 2017). EEG data were re-referenced to average reference, and the mean amplitude of the 100 ms baseline interval was subtracted at all time points on each channel. Only trials including a correct response, given within the 1,000 ms interval after stimulus onset, were retained. On average, 84 target trials per participant were retained in 2-rule runs (SD = 14.8), and 82 target trials per participant were retained in 4-rule runs (SD = 14.2). For lures, 94 trials per participant were retained in 2-rule runs (SD = 2.3), and 94 trials per participant were retained in 4-rule runs (SD = 1.8). For filler, 1,148 trials per participant were retained in 2-rule runs (SD = 8.9), and 1,147 trials per participant were retained in 4-rule runs (SD = 9.4).

### Source Analyses

Source estimates were derived from EEG data by using the MNE Python software v0.21.0 package in combination with FreeSurfer (Version 6).^2^ A minimum norm estimation (MNE) method (Hämäläinen and Ilmoniemi, 1994; Hauk, 2004) was used that makes minimal *a priori* assumptions on the distribution of cortical sources. EEG sensor configurations were co-registered with a high-resolution structural T1-weighted MRI image implemented in the MNE Python software, using digitizer points from a standard montage template. The scalp surface of the MRI image was reconstructed by using the automated segmentation algorithms of the FreeSurfer software. By using the traditional method for cortical surface decimation, the original triangulated cortical surface (consisting of several hundred thousand vertices) was down-sampled to a grid with an average distance between vertices of 5 mm, which resulted in approximately 1,000 vertices. The boundary element model was created with three layers for scalp, outer skull surface, and inner skull surface. Dipole sources were assumed to be almost perpendicular to the cortical surface, with some variation in the tangential plane (one fifth of the radial dimension). Note that source distribution time courses are unaffected by the choice of EEG reference electrode. This is one of the advantages of source space compared with scalp EEG analysis.

Source estimates were computed for each participant and condition (targets, lures, fillers, and cues for 2- and 4-rule runs, respectively). Time windows of interest for statistical analyses on EEG source estimates were defined based on the peaks in mean EEG source activity across all vertices and conditions of interest (targets, lures, and filler). Cues were excluded from the main analysis due to their small number of trials. Only correct responses were included in data analyses. Responses to targets were considered correct if they occurred within 1,000 m following target onset (i.e., before onset of the following stimulus); responses to cues, lures, and fillers were considered correct if there was no keypress in this time period. To eliminate chunks where participants may have missed a cue or searched for the wrong target, all targets and lures for a particular chunk were removed if no response was given to either of the two targets, an equal number of responses were given to targets and lures, or more responses were given to lures than to targets (Tschentscher et al., 2017).

Signal from predefined regions of interest (ROIs) of the MD network (Duncan, 2010; Tschentscher et al., 2017) was extracted using two bilateral frontal and two bilateral parietal parcellations of the cortical surface created by the neuroanatomical labels of the Freesurfer software. We aimed for the ROIs capturing executive control effects in regions of the MD network (Duncan, 2010), as observed in the fMRI study by Tschentscher et al. (2017), which used a very similar task design and observed individual differences in fluid intelligence in a frontal-parietal network including the ventro-lateral and orbito-medial prefrontal cortex, as well as the posterior superior parietal lobule and angular gyrus. These regions were covered by our frontal and parietal ROI labels, respectively. However, we cannot be absolutely certain that the peaks of activity we see in our EEG source estimates correspond exactly to peaks in fMRI activation from previous studies. For this reason, whole-brain distributions of MNE source estimates were obtained for those contrasts that showed significant effects in ROI analyses, to explore those putative effects outside of our pre-defined ROIs.

Time windows for statistical analyses (200–300 and 300–500 ms) were defined based on mean signal intensity across all vertices in source space for the average across all conditions of interest (targets, lures, and fillers), as well as based on previous EEG sensor level studies that reported early effects of individual differences in executive functions and fluid intelligence from 200 ms post stimulus onset (Brumback et al., 2004; Rueda-Delgado et al., 2019; Luo and Zhou, 2020).

## Results

### Behaviour

**FIGURE 2:**
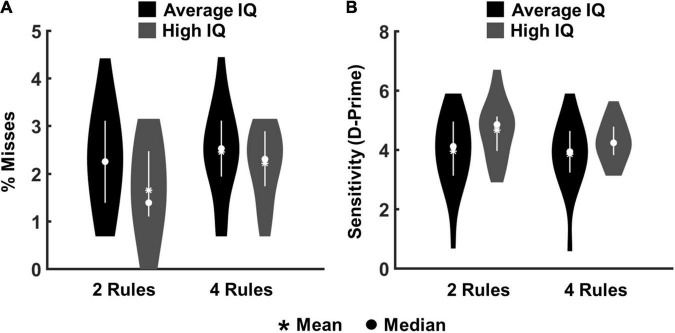
Behavioural results from the eight main chunks of all experimental runs for high-IQ and average-IQ participants. **(A)** Percentages of misses for targets. **(B)** Sensitivity index (d-prime) reflecting the proportion of correct responses (hits) in relation to false alarms.

Data from 40 participants entered the analysis. For all participants rule learning, as measured by the retrieval-task in the beginning and end of each run, reached 100% accuracy. Our primary behavioural analyses concerned just the eight main chunks of each run, examining misses to targets (no key-press within 1,000 ms of stimulus onset) and false alarms to lures (key-press in corresponding interval). The impact of task complexity and IQ was assessed by applying an ANOVA with the factors “complexity” (2- vs. 4-rules) and “IQ group” (high-IQ vs. average-IQ). For misses, the Kolmogorow-Smirnow-Lilliefors test was used to test for normality assumption in each of the four conditions (high-IQ and average-IQ groups on each complexity level). The data of each condition was log-transformed for statistical analyses due to violation of normality assumptions in two of the four conditions. Statistical analyses were performed on averages over trials within each condition. There was a significant main effect of complexity [*F*(1,38) = 17.25, *p* < 0.001, η^2^ = 0.312], as well as a significant complexity × IQ group interaction [*F*(1,38) = 5.79, *p* = 0.021, η^2^ = 0.132], which was driven by highest percentages of misses in the average-IQ group during 4-rule runs (mean percentage of misses = 2.45), and lowest percentages of misses in the high-IQ group during 2-rule runs (mean percentage of misses = 1.64) (Figure 2A). The main effect of IQ group was not significant (*p* = 0.133).

To evaluate the proportion of correct responses (hits) in relation to false alarms, we also calculated the sensitivity or d’ index [referring to d’ = Z(hit rate) − Z(false alarm rate); Macmillan and Creelman (2005)]. This is a measure of overall performance accuracy (i.e., how well participants distinguished between the two critical stimulus events; targets and lures). The Kolmogorow-Smirnow-Lilliefors test was used to test for normal distribution in each of the four conditions (i.e., high-IQ and average-IQ groups on each complexity level). No significant deviation from normal distribution was observed in any condition. The analysis confirmed the main effect of complexity [*F*(1,38) = 16.65, *p* < 0.001, η^2^ = 0.304] as well as the complexity × IQ group interaction [*F*(1,38) = 6.68, *p* = 0.013, η^2^ = 0.149], which was driven by the lowest d-prime score in the average-IQ group during 4-rule runs (mean d-prime score = 3.84), and the highest d-prime score in the high-IQ group during 2-rule runs (mean d-prime score = 4.65). The main effect of IQ group was not significant (*p* = 0.087; Figure 2B). The overall percentages of misses and false alarms were low, ranging between 10 and 15 percent across participants, indicating good overall ability to follow task instructions.

We also explored the percentage of false alarms to filler items. The data of each condition was log-transformed for statistical analyses due to violation of normality assumptions in three of the four conditions. The overall number of false alarms to filler items was below one percent in both average-IQ and high-IQ groups. There was a significant main effect of complexity [*F*(1,38) = 6.68, *p* < 0.014, η^2^ = 0.149], but no significant interaction with IQ group (*p* = 0.088) and no significant main effect of IQ group (*p* = 0.068).

To ensure that all participants adequately considered all four rules in the 4-rule condition, we also examined misses and false alarms in the brief task periods devoted to the 3rd and 4th rules (Figure 1). The number of misses was low, and did not differ significantly between IQ groups [mean number of misses for average-IQ = 1.8, SD = 2.0; mean number of misses for high-IQ = 1.2, SD = 1.15; *t*(38) = 0.921, *p* = 0.362]. There were no false alarms to lures in the brief task periods devoted to the 3rd and 4th rule. The absence of significant IQ group effects might reflect the lack of statistical power inherent in analyses of the 3rd and 4th rules, since those rules only appeared twice within each 4-rule run. Overall, the results confirm that 3rd and 4th rules, like the two rules in the main chunks that were used for comparisons of 2- and 4-rule runs, were learned and followed.

### Electroencephalography

**FIGURE 3:**
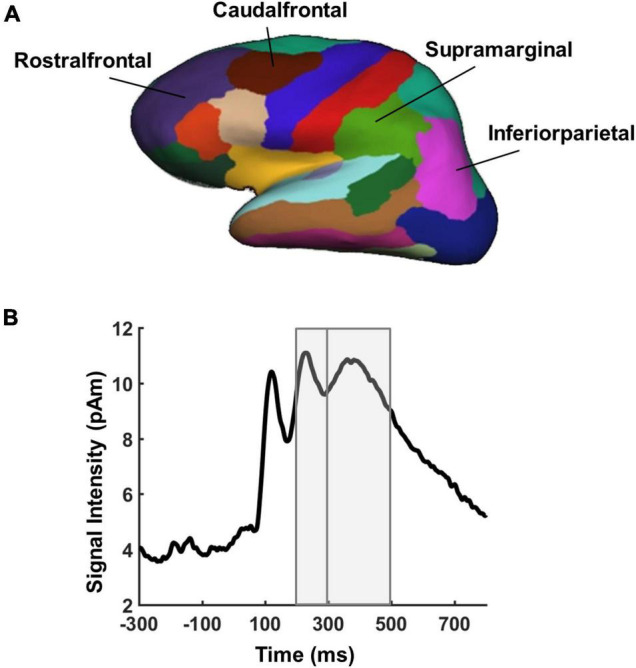
Regions and time windows of interest for EEG source analyses. **(A)** Bilateral frontal and bilateral parietal parcellations of the cortical surface created by neuroanatomical labels of the Freesurfer software based on the Desikan-Killiany Atlas (Desikan et al., 2006). **(B)** Overall activation time courses in evoked source estimates averaged across all vertices and task conditions of interest (targets, lures, and fillers). Gray areas indicate the time windows used for statistical analyses.

We assessed the recruitment of the MD network during performance of the task by extracting mean evoked source estimates from four predefined ROIs within each hemisphere based on parcellations of the Freesurfer software (supramarginal, inferiorparietal, rostralfrontal, and caudalfrontal, see Figure 3A) using the Desikan-Killiany Atlas (Desikan et al., 2006). Our main interest concerned the impact of IQ and task complexity on the processing of targets and lures. Two time windows were defined based on mean signal intensity across all vertices in source space for the average across target, lure, and filler conditions, and in line with early effects of individual differences in executive control and fluid intelligence from previous EEG sensor level studies (Brydges et al., 2014; Amin et al., 2015; Luo and Zhou, 2020; Figure 3B): 200–300 and 300–500 ms.

Average source estimate amplitudes were computed within time windows and across vertices inside each of the ROIs. These were subjected to an ANOVA with the factors “complexity” (2- vs. 4-rules), “IQ group” (average-IQ vs. high-IQ), “condition” (targets, lures, and fillers), “time window” (200–300 vs. 300–500 ms), and “ROI” (caudalfrontal L/R, rostralfrontal L/R, supramaginal L/R, and inferiorparietal L/R). For significant interactions between the factors “condition,” “complexity,”and “IQ group”, *post-hoc* ANOVAs were conducted including the factors “complexity” (2- vs. 4-rules) and “IQ group” (average-IQ vs. high-IQ). The Kolmogorow-Smirnow-Lilliefors test was used to test for normality assumption in each of the four conditions (high- and average-IQ groups on each complexity level). The data of each condition was log-transformed for statistical analyses due to violation of normality assumptions.

No significant interaction between the factor “condition” (target, lures, and filler) and the factors “complexity” and “IQ group” was observed, suggesting a similar response pattern for targets, lures, and fillers. A significant IQ group × ROI interaction [*F*(7,266) = 4.97, *p* < 0.001, η^2^ = 0.116], a significant complexity × IQ group × ROI interaction [*F*(7,266) = 2.11, *p* = 0.043, η^2^ = 0.052], a significant time window × IQ group × ROI interaction [*F*(7,266) = 2.71, *p* = 0.010, η^2^ = 0.066], and a significant complexity × ROI × time window interaction [*F*(7,266) = 3.75, *p* = 0.001, η^2^ = 0.090] was observed, suggesting that an interaction between the factors “IQ group” and “complexity” varied for time windows as well as ROIs. The responses for targets, lures, and fillers were averaged and ANOVAs with the factors “complexity” and “IQ group” were run for each time window as well as ROI separately.

**FIGURE 4:**
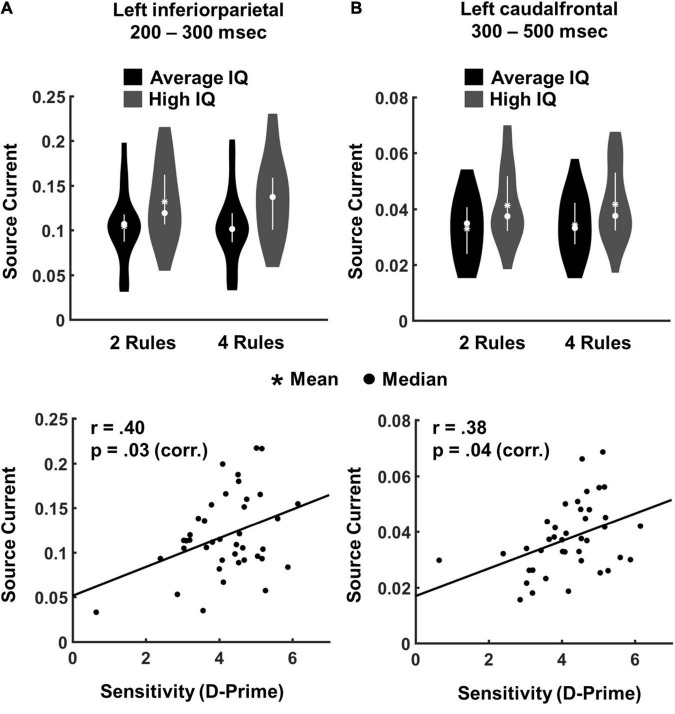
Region of interest analyses in pre-defined time windows. **(A)** The plot shows EEG source current indicating the significant main effect of IQ group in left inferiorparietal cortex in the 200–300 ms time window, and the significant correlation between EEG source current and task performance (d’ scores). **(B)** EEG source current indicating the significant main effect of IQ group in left caudalfrontal cortex in the 300–500 ms time window, and the significant correlation between EEG source current and task performance (d’ scores).

For the 200–300 ms time window, significant main effects of IQ group were observed in left inferioparietal cortex [*F*(1,38) = 5.03, *p* = 0.031, η^2^ = 0.116], right inferiorparietal cortex [*F*(1,38) = 11.53, *p* = 0.001, η^2^ = 0.232] and right supramarginal cortex [*F*(1,38) = 7.74, *p* = 0.008, η^2^ = 0.169]. No significant effect of complexity or interaction with IQ group was observed. Source estimates for each of these ROIs were averaged across complexity conditions and subjected to brain-behaviour analyses: Bonferroni corrected correlations between source estimates from these ROIs and measures of performance accuracy (d’ indices averaged across levels of complexity) revealed that higher source amplitudes in left inferiorparietal cortex were associated with better task performance (*r* = 0.401, *p* = 0.030 Bonferroni corrected by three ROIs) (Figure 4A).

For the 300–500 ms time window, a significant main effect of IQ group was observed in left caudalfrontal cortex [*F*(1,38) = 4.28, *p* = 0.044, η^2^ = 0.101], and in right inferior parietal cortex [*F*(1,38) = 7.31, *p* = 0.010, η^2^ = 0.161]. A main effect of complexity occurred in right inferiorparietal cortex [IQ *F*(1,38) = 11.23, *p* = 0.001, η^2^ = 0.228]. In order to address putative effects of lateralization in frontal ROIs, we additionally performed a Bayesian Repeated Measure ANOVA with the factors “complexity” (2- vs. 4-rules), “IQ group” (average-IQ vs. high-IQ) on source current extracted from the right caudalfrontal cortex in the 300–500 ms time window. A BF_01_ of 1.54 suggested weak evidence for the null hypothesis concerning the main effect of “IQ group.” Again, sources estimates for each of these two ROIs were averaged across complexity conditions and subjected to brain-behaviour analyses: Bonferroni corrected correlations between source estimates from these ROIs and measures of performance accuracy (d’ indices averaged across levels of complexity) revealed that higher source amplitudes in left caudalfrontal cortex were associated with better task performance (*r* = 0.377, *p* = 0.048 Bonferroni corrected by three ROIs; Figure 4B).

Exploratory analyses of the cue trials did not reveal any significant effect in ANOVAs with the factors “complexity” and “IQ group” within the pre-defined ROIs and time windows.

**FIGURE 5:**
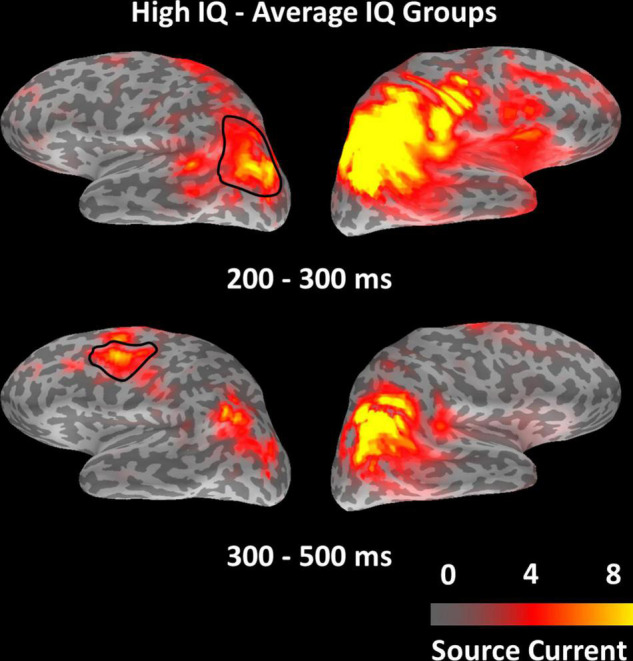
Distribution of test statistic values for the contrast between IQ groups (high-IQ – average-IQ) from the whole-brain non-parametric cluster-level test for spatio-temporal data, displayed for the two windows of 200–300 and 300–500 ms, respectively. The highlighted left-hemispheric parcellations indicate ROIs in which higher source amplitude levels in the high-IQ group were associated with better task performance, in contrast to the average-IQ group (cf. correlation analyses of Figure 4).

To check for activity outside of a-priori defined ROIs for the observed main effects of IQ group, we ran a whole-brain non-parametric two-sample cluster-level test for spatio-temporal data (Maris and Oostenveld, 2007) on EEG minimum norm estimates, including the conditions of targets, lures, and fillers. After correction for multiple comparisons, no significant IQ group effect was observed at the whole-brain level. To make sure our initial approach of *a priori* selecting ROIs did not lead us to miss any important effects, whole-brain analyses were re-run at a lenient threshold of *p*(un-corrected) = 0.030, which was the significance level of the earliest IQ group effect in ROI analyses. Effects of IQ group only appeared within or adjacent to regions of the MD network, as used in our ROI analysis (Figure 5). The result confirms that MD network regions were most affected by our experimental manipulations, and it indicates that the selected *a priori* defined ROIs captured any IQ effects well.

## Discussion

This study addressed the spatio-temporal neural dynamics of human fluid intelligence. The results link individual differences in fluid intelligence to an early process of attention focus on task-critical information, supported by dorsal MD network regions. In our paradigm, novel rules have been instructed across experimental runs (either 2 or 4 rules). Within each run, only one rule was cued at a time in a continuous sequence of stimuli. Within 4-rule runs, only 2 out of 4 rules were cued at those chunks that were used for comparisons of 2- and 4-rule runs. Thus, although the number of instructed rules varied across runs, the complexity of each task period remained constant. Knowledge of rules was assessed in the beginning and end of each run. Participants from average-IQ and high-IQ groups were instructed to respond to the cued target and to ignore the images associated with a different cue (lure), as well as those never associated with any cue (fillers).

First individual differences in fluid intelligence emerged in evoked EEG source estimates in bilateral inferiorparietal cortices in early time-windows of task processing from 200 ms onward, as well as in left caudalfrontal cortex at a slightly later time window, from 300 ms onward. Participants of higher fluid intelligence showed stronger neural signal in these regions. Whole-brain source analyses confirmed the localization of these effects within, or adjacent to, regions of the MD network: early individual differences in fluid intelligence were observed in bilateral parietal MD regions in the 200–300 ms time window. Subsequent individual differences in fluid intelligence were observed in left-hemispheric frontal MD regions in the 300–500 ms time window. This indicates that MD regions do not operate in parallel across time, as it might have been discussed based on fMRI research, but rather show a differential activation pattern in early and later phases of cognitive processing. For the first time, this study provides spatio-temporal neural signatures of fluid intelligence from MD regions, with a millisecond temporal resolution and good anatomical precision within centimetre-range. Crucially, individual differences in fluid intelligence did emerge already in early phases of rule selection from 200 ms onward, in which the rule in operation had to be foregrounded from the memorised task set. However, concerning the putative lateralization of frontal MD region effects in the 300–500 ms time window, it is important to note that the absence of individual differences in fluid intelligence in right frontal MD region might be due to lack of statistical power rather than hemispheric-asymmetries within the MD network. Furthermore, we cannot rule out that any other cognitive mechanism related to fluid intelligence, or even unrelated to fluid intelligence, i.e., salience or attention (Friston, 2009), has contributed to the discussed neural activation pattern in the MD network.

In brain-behaviour correlation analyses, earliest neural differences of intelligence groups in left inferiorparietal cortex also predicted participants’ performance in rule use, i.e., their ability to foreground the correct rule at a given point in time from the memorised task set. This indicates the specific role of this brain region in fluid intelligence, as it seems to support an early process of attentional focus on critical task rules. Thus, our results on the spatio-temporal neural dynamics specify the role of left-hemispheric parietal MD regions in early cognitive processes underlying fluid intelligence. This highlights the importance of analysing time-resolved neural correlates of individual differences in fluid intelligence, while previous fMRI studies reported spatially indifferent responses as a function of fluid intelligence across the whole MD network (Duncan and Owen, 2000; Tschentscher et al., 2017).

On the behavioural level, our results are consistent with previous research reporting a phenomenon of goal neglect in individuals with lower fluid intelligence (Duncan et al., 2008; Bhandari and Duncan, 2014). In goal neglect, task requirements are repeatedly ignored during performance, although participants accurately describe them before and after testing. We observed a main effect of complexity (2- vs. 4-rule runs) in task accuracy, as well as an interaction between fluid intelligence (average-IQ vs. high-IQ groups) and complexity. This behavioural interaction effect reflects the particular difficulty of average-IQ participants in foregrounding and implementing the specific rule of the current task period, when the rule was to be drawn from a larger overall set.

We here add crucial information on the spatio-temporal neural dynamics of fluid intelligence to the results from our recent fMRI study using the same task paradigm (Tschentscher et al., 2017): in both studies we observed higher percentages of errors in behavioural performance for average-IQ participants during 4-rule runs. Moreover, we replicated the weaker neural responses of average-IQ participants for task-relevant stimuli in regions of the MD system, confirming our previous claim that weak recruitment in lower-ability individuals reflects poor attentional focus, or poor discrimination of critical events from the ongoing task background (cf. Tschentscher et al., 2017). Interestingly though, in the current study we did not observe a significant effect of task complexity on the neural level, in contrast to the previous fMRI study, while we replicated effects of task complexity in behavioural performance measures. The absence of task complexity effects on the neural level might be due to overall differences in mean IQ scores across studies, which were higher in the current study in both average-IQ and high-IQ groups. It might be also due to the precise temporal resolution of neural responses in the current study, which was able to differentiate between individual stages of cognitive processing, i.e., between early processes of attentional focus and later rule execution, in contrast to the previous fMRI research.

Our data suggest that individual differences in fluid intelligence are linked to a process of attentional focus on task-critical information in early time windows from 200 ms onward. As such, these results are related well with previous evoked EEG analyses at the sensor level, suggesting that early individual differences within 200–300 ms time windows are in particular predictive for levels of executive control and fluid intelligence in children and adult populations (Wronka et al., 2013; Brydges et al., 2014; Amin et al., 2015; Luo and Zhou, 2020). These studies reported stronger amplitudes about 300 ms post stimulus-onset during an oddball task in adult participants with higher scores on the Raven’s Advanced Progressive Matrices test (Wronka et al., 2013), as well as during a learning and memory task in higher-intelligent adult participants (Amin et al., 2015). Higher amplitudes and a shorter latency of effects were also observed in 200–300 ms windows during a working memory task in highly intelligent children (Luo and Zhou, 2020). Moreover, our results are consistent with previous EEG studies linking individual differences in fluid intelligence to a parietal component of the P3-family (P3b component) that has been associated with rule learning and the allocation of attentional resources to task relevant features (Amin et al., 2015; Teixeira-Santos et al., 2020). We here replicate individual differences in fluid intelligence in EEG sources analyses within the same time windows, and for the first time link these effects to specific neural responses in left parietal and left caudalfrontal regions, respectively.

In our data, the left inferiorparietal cortex is linked with earliest cognitive processes underlying individual differences in fluid intelligence. The inferiorparietal cortex is part of the dorsal attention network, as well as the MD system (Yeo et al., 2011; Santarnecchi et al., 2017b). It has been previously associated with visual-spatial attention and attentional orientation, as well as with the core processes underlying executive control (Clark et al., 2017). By using graph theory and meta-analytic functional profiling, it has been claimed that the frontoparietal executive control network may exert top-down control over the dorsal attention network to ensure that attention remains focused on task-relevant information instead of distracting stimuli (Dixon et al., 2018). This might explain why earliest effects as a function of fluid intelligence occurred in parietal regions in the current study: the complex target detection task required the foregrounding and implementation of a specific rule (the target in operation) over the course of a task chunk, while distracting information (lures, i.e., the targets not in operation) had to be cancelled out. Our data suggest that in particular the left dorsal part of the MD network - putatively in interaction with the dorsal attention network - is crucial for this process, by linking stronger early neural responses in this region to high fluid intelligence, and a process of attentional orientation to task-relevant features.

To conclude, this study provides novel evidence on the specific neural signatures of individual MD regions across the time-course of complex cognitive processing. Specifically, we here show that, amongst all MD system regions, the left parietal cortex is in particular crucial for earliest processes underlying individual differences in fluid intelligence: the allocation of attentional resources to those parts of a task that are most critical for behaviour.

## Data Availability Statement

The raw data supporting the conclusions of this article will be made available by the authors, without undue reservation.

## Ethics Statement

The studies involving human participants were reviewed and approved by Ethikkommission der Fakultät für Psychologie und Pädagogik der Ludwig-Maximilians-Universität München. The patients/participants provided their written informed consent to participate in this study.

## Author Contributions

NT performed the research, analyzed the data, and wrote the first draft of the manuscript. NT and PS designed the research, edited the manuscript, and approved the submitted version.

## Conflict of Interest

The authors declare that the research was conducted in the absence of any commercial or financial relationships that could be construed as a potential conflict of interest.

## Publisher’s Note

All claims expressed in this article are solely those of the authors and do not necessarily represent those of their affiliated organizations, or those of the publisher, the editors and the reviewers. Any product that may be evaluated in this article, or claim that may be made by its manufacturer, is not guaranteed or endorsed by the publisher.

## Footnotes

1
http://psychtoolbox.org/

2
surfer.nmr.mgh.harvard.edu/
